# Incidence of School Failure According to Baseline Leisure-Time Physical Activity Practice: Prospective Study

**DOI:** 10.1016/j.jadohealth.2012.06.024

**Published:** 2012-12

**Authors:** Airton J. Rombaldi, Valerie L. Clark, Felipe F. Reichert, Cora L.P. Araújo, Maria C. Assunção, Ana M.B. Menezes, Bernardo L. Horta, Pedro C. Hallal

**Affiliations:** a Postgraduate Program in Epidemiology, Universidade Federal de Pelotas, Pelotas, Brazil; b Physical Activity Epidemiology Research Group, Universidade Federal de Pelotas, Pelotas, Brazil; c Postgraduate Program in Physical Education, Universidade Federal de Pelotas, Pelotas, Brazil

**Keywords:** School achievement, Adolescents, Cohort, Physical activity

## Abstract

**Purpose:**

To evaluate the prospective association between leisure-time physical activity practice at 11 years of age and incidence of school failure from 11 to 15 years of age.

**Methods:**

The sample comprised >4,300 adolescents followed up from birth to 15 years of age participating in a birth cohort study in Pelotas, Brazil. The incidence of school failure from age 11 to 15 years was calculated by first excluding from the analyses all subjects who experienced a school failure before 11 years of age, and then categorizing as “positive” all those who reported repeating a grade at school from 11 to 15 years of age. Leisure-time physical activity was measured using a validated questionnaire.

**Results:**

The incidence of school failure was 47.9% among boys and 38.2% among girls. Adolescents in the top quartile of leisure-time physical activity practice at 11 years of age had a higher likelihood of school failure (OR: 1.36; 95% CI: 1.06, 1.75) compared with the least active adolescents. In adjusted analyses stratified by sex, boys in the top quartile of leisure-time physical activity practice at 11 years of age were also more likely to have failed at school from age 11 to 15 years (OR: 1.60; 95% CI: 1.09, 2.33).

**Conclusions:**

Adolescents allocating >1,000 min/wk to leisure-time physical activity were more likely to experience a school failure from 11 to 15 years of age. Although this finding does not advocate against physical activity promotion, it indicates that excess time allocated to physical activity may jeopardize school performance among adolescents.

**Implications and Contribution**

Although adolescent physical activity provides several short and long-term benefits for health, adolescents allocating 2.5 hr/d to leisure-time physical activity practice are more likely to experience school failures, thus suggesting that excess time allocated to physical activity may jeopardize school performance.

Regular physical activity in adolescence has both short- and long-term benefits for health, including reducing the risk of being overweight, obesity, and many diseases [1,2]. In the long-term, bones and skeletal muscles of active adolescents become stronger owing to increases in muscular strength and resistance as compared with inactive adolescents [3]. Also, physical activity in adolescence relates to positive self-esteem and the reduction of stress and anxiety [1]. Besides known physiological effects resulting from physical activity practice, there is evidence that fitness may influence academic achievement in children and adolescents [4,5]. Experimental studies have demonstrated that physical activity interventions might be used to improve cognitive and behavioral functions, especially in children [6,7]. The hypothesis considering the existence of this association is old, but its systematic investigation is relatively recent. However, most studies used a cross-sectional design to test this association, thus limiting our understanding of causality [8,9].

A possible association between physical activity indicators and school performance might be because of two different explanations. First, if physical activity biologically results in better cognitive performance, a positive association between physical activity and school performance is to be expected. On the other hand, excess time dedicated to physical activity may damage academic performance [10–12], not through a biological mechanism, but simply owing to lack of time for studying.

The aim of our study was to evaluate the prospective association between leisure-time physical activity at 11 years of age and incidence of school failure from 11 to 15 years of age. Our primary hypothesis was that physical activity practice within the recommended level of 300 min/wk will not have a detrimental impact on school performance throughout adolescence.

## Methods

Pelotas (population 320,000 inhabitants) is located in southern Brazil in a relatively developed part of the country. In 1993, a birth cohort study was initiated in the city. Mothers of all hospital-born children, representing >99% of all deliveries in Pelotas, (n = 5,265) were invited to join the study. More than 99% (n = 5,249) of mothers agreed to participate in the study. Since 1993, the national mortality system has been regularly monitored, and deaths of cohort members are linked to the study database. Mothers were interviewed soon after delivery on family characteristics, gestation-related variables, and behavior. Socioeconomic status was evaluated using an assets index. It included questions on the possession (and quantity) of household assets, such as television, radio, air conditioning, phone lines, fridge, among others, and a standardized procedure was used to generate a continuous score through principal component analysis that was later divided into quintiles. Parental education was expressed in years of schooling.

In 2004–2005, we conducted a follow-up visit to all participants of the cohort. We were able to locate 4,452 participants, which represent 87.5% of the cohort taking into account the 141 subjects known to have died. In 2008, another wave of data collection was conducted, and we were able to locate 4,325 cohort members. In both visits, participants were reimbursed for any transportation or food expenses, but no other incentives were given for taking part in the follow-up visits. A detailed description of the cohort has been published elsewhere [13].

Transport and leisure-time physical activity were measured using a validated questionnaire [14]. The questionnaire initially asks about the primary mode of transportation to and from school. A list of several culturally relevant leisure-time physical activities, including sports, was later shown to participants, and they were asked whether they have practiced those activities over the past week. For each positive answer, information on weekly frequency and duration was obtained. Frequency was multiplied by time, generating a weekly score for each activity; later the scores were summed, producing a leisure-time physical activity score. For this analysis, we opted to ignore commuting time for three main reasons: (a) a large proportion of total physical activity in this age-group in Brazil is practiced in the transportation to and from school [14]; (b) transport-related physical activity at this age is much more a matter of poverty than a choice in Brazil [15]; and (c) the intensity of walking or cycling to school tends to be low [16]. The continuous score of leisure-time physical activity was transformed into quartiles for the analysis.

The reliability and concurrent validity of the physical activity questionnaire were tested in a previous study [14]. Its reliability was good (rho: .62; *p* < .001); 73% of the subjects were classified consistently in a 7-day test–retest exercise. The kappa value was .58. The concurrent validity of the questionnaire was tested against pedometers; the Spearman correlation coefficient was .26 (*p* = .02), and 57% of the subjects were classified consistently as physically inactive in the questionnaire and with pedometers (using a cutoff point of 10,000 steps/d).

School failure was defined as having ever repeated a grade at school. We calculated the incidence of school failure from age 11 to 15 years by first excluding from the analyses all subjects who experienced a school failure before 11 years of age, and then categorizing as “positive” all those who reported failing at school from age 11 to 15 years. This strategy was used owing to the basic cohort study principle that subjects should be disease-free at baseline.

School failure was analyzed as a dichotomous variable (yes vs. no). Confounding variables were sex, parental schooling, family assets index, type of school, age at school entry, and child labor (yes vs. no). These variables were selected because the literature search indicated a possible association with both the outcome and the exposure variable.

Analyses were conducted using Stata 11.0 (StataCorp LP, College Station, TX). First, we calculated the proportion of school failure according to quartiles of maternal and paternal schooling. Second, we explored the association between physical activity and socioeconomic position. These analyses were carried out to understand the confounding structure of our data. The main analyses included estimating odds ratios for having experienced a school failure between 11 and 15 years of age according to leisure-time physical activity indicators at 11 years of age. Finally, we stratified the association between sports practice and school failure by gender due to a significant interaction.

All phases of the 1993 Pelotas (Brazil) Birth Cohort Study obtained institutional review board approval. Written informed consent was signed by mothers or guardians in all visits, and verbal consent was given by adolescents at the 11- and 15-year-old follow-up visits.

## Results

Individuals followed at 11 and 15 years (N = 4,317) of age were similar to the remaining cohort members (N = 932) in terms of sex and parental schooling. At the age 15-year follow-up visit, 48.9% of the subjects were male, 22% had a paid job over the past 12 months, 18% had ever smoked, and 8% were obese. Of 4,410 cohort members with data on schooling levels at 11 years of age, 1,653 (37.5%) had already experienced school failure at that age. These individuals were therefore excluded from the prospective analysis on the incidence of school failure. Of the remaining 2,757 participants, we were able to locate 2,657 at 15 years of age. From the latter, 1,128 (42.5%) experienced school failure between 11 and 15 years of age.

**Figure 1:**
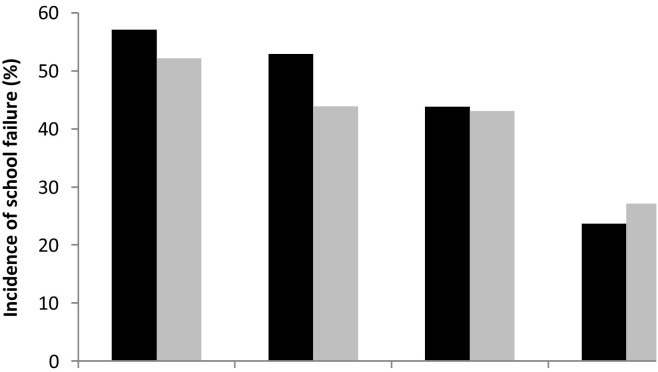
Incidence of school failure according to quartiles of parental schooling.

**Figure 2:**
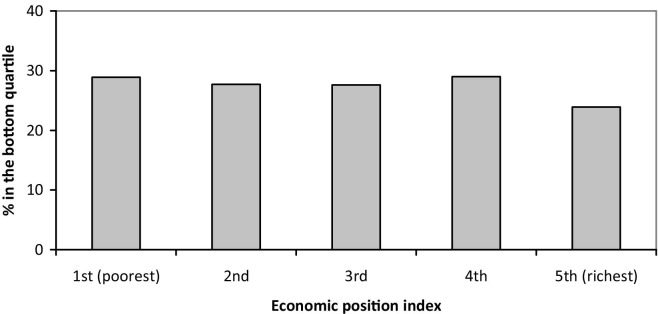
Proportion of adolescents in the least active quartile of leisure-time physical activity according to quintiles of the assets index.

The incidence of school failure was 47.9% among boys and 38.2% among girls. Parental schooling at birth was linearly related to the incidence of school failure in adolescence (Figure 1). Figure 2 presents the proportion of adolescents classified in the least active group (bottom quartile) of leisure-time physical activity according to quintiles of the family assets index. No clear association was found between leisure-time physical activity and quintiles of the assets index.

Table 1 presents the unadjusted and adjusted associations between baseline leisure-time physical activity and incidence of school failure. In the unadjusted analyses, there was a dose–response positive association between leisure-time physical activity and school failure. After adjustment for confounders, especially socioeconomic indicators, the trend was no longer observed. However, those in the top quartile of leisure-time physical activity at 11 years of age have a higher likelihood of school failure (OR: 1.36; 95% CI: 1.06, 1.75).

Table 2 presents the adjusted associations between baseline leisure-time physical activity and incidence of school failure according to sex. After adjustment for the confounders, socioeconomic indicators and parental schooling, boys in the top quartile of leisure-time physical activity at 11 years of age were more likely to fail at school between 11 and 15 years of age (OR: 1.60; 95% CI: 1.09, 2.33).

## Discussion

Evaluating the association between physical activity and school performance is challenging owing to the complex confounding structure. Parental schooling is one of the most important determinants of academic achievement. At the same time, total physical activity is more frequent among those whose parents have lower education levels because of higher engagement in commuting activities. Therefore, it is expected that in the unadjusted analyses using total physical activity as the exposure, those more active (whose parents are less educated) will be more likely to have experienced failures at school, in part owing to their parents' lower schooling levels. This is why we decided not to analyze total physical activity and instead focus on leisure-time physical activity only. Our comprehensive adjustment for socioeconomic indicators (e.g., parental schooling, assets index) eliminates part of the possible confounding role of socioeconomic position on the association investigated here. Nevertheless, the possibility of residual confounding cannot be ruled out. Also, other variables that might affect school repetition were not addressed, such as type of activities performed, cultural values and norms, peer influences, among others [16].

Previous research has reported a relationship between physical activity indicators and learning or academic success [4–7,17–19] among children and adolescents. Studies indicated that replacing academic learning sessions with physical activity did not have a detrimental impact on school performance [20,21]. Evidence has also suggested that achieving a threshold amount of physical activity may be necessary to acquire learning benefits [22], and that participation in vigorous-intensity physical activity may further enhance learning [23].

Other studies have found that participation in sports has a positive association with academic outcomes, such as higher grade point averages in both boys and girls in early and late adolescence, higher retention rates, and less absenteeism, among other predictors of success in school [24–27].

Our study showed that leisure-time physical activity practice >1,000 min/wk increased the incidence of school failure by 36%; this association was stronger in boys than in girls. This finding points to time allocation as a determinant of school achievements. Our top quartile had a mean daily allocation of 2.5 hours of leisure-time physical activity. If we assume that around 4 hr/d are spent at school in Brazil, then 6.5 hours of the day are scheduled. When time spent in front of screens (approximately 5 hr/d in this cohort) (Dumith S.C., et al, unpublished data, 2011), sleeping (approximately 8 hr/d in this cohort) [28], and eating are added, it is possible these individuals did not have adequate time to study. Furthermore, because of the lack of measurement of cognitive performance in these adolescents, our findings should not be interpreted as evidence of a negative influence of leisure-time physical activity on cognition. In fact, it seems time allocation could be the main explanation for our findings.

Previous studies have found analogous results [10,11,29]. One of these studies, for example, showed that the need for more study time was the main reason for sports dropout [10]. Another study suggested that school teachers tried to create mechanisms to reduce the academic thresholds for those involved in sports, clearly assuming that adolescents practicing sports would be unable to achieve standard school performance [11].

An important methodological aspect to be considered is the fact that previous research often asks about physical activity or sports participation dichotomously, and therefore could not evaluate the role of the tail of the distribution (i.e., excessive physical activity practice). In fact, if our variable were dichotomous, we would also fail to find associations because the first three quartiles did not differ significantly from each other. Therefore, we suggest that future studies in this field include continuous variables about leisure-time physical activity so the different extremes of the variables can be analyzed separately.

Our findings do not refute a possible biological association between physical activity practice and enhanced cognitive ability. Previous studies have suggested that participation in different sources of physical activity, including physical education, physical activity in recess time, and sports practice [24,30–34], is likely to benefit academic performance and include improvements in the acquisition of motor abilities. Our article adds to this knowledge by showing that there is likely a threshold for that, and excess time spent on physical activity can also have a detrimental effect.

In summary, adolescents allocating >1,000 min/wk to leisure-time physical activity are more likely to experience a school failure from 11 to 15 years of age. This finding has important public health consequences. Adopting policies similar to the “No Pass, No Play” rule currently used in several places in the United States could be a tactic to overcome the problem detected in our analyses of Brazilian adolescents. Although approaches aimed at restricting physical activity participation based on academic achievement have some negative consequences [35], this type of strategy has also been shown to improve academic outcomes, such as the reduction of school dropout rates and increased graduation rates [36]. Care should be exercised before extrapolating our findings to other settings, particularly because in Brazil adolescents typically spend only 4 hours in school, which is different from other parts of the world. However, even if the threshold happens to be different in other countries, it would be important to test whether those allocating too much time in leisure-time physical activity will have worst academic performance.

## Figures and Tables

**Table 1:** Unadjusted and adjusted associations between leisure-time physical activity at 11 years of age and incidence of school failure from 11 to 15 years

Leisure-time physical activity practice at 11 years (quartiles)	Unadjusted analyses		Adjusted analyses^a^	
	OR (95% CI)	*p*	OR (95% CI)	*p*
1st (mean 5.8 min/wk)	1.00		1.00	
2nd (mean 92.8 min/wk)	1.04 (.84, 1.29)	.71	1.11 (.87, 1.41)	.40
3rd (mean 247.5 min/wk)	1.07 (.86, 1.33)	.54	1.11 (.87, 1.43)	.40
4th (mean 1,009.0 min/wk)	1.27 (1.02, 1.58)	.03	1.36 (1.06, 1.75)	.01

CI = confidence interval; OR = odds ratio.

a Adjusted for sex, maternal and paternal schooling, and family assets index.

**Table 2:** Adjusted associations between leisure-time physical activity at 11 years of age and incidence of school failure from 11 to 15 years according to sex

Leisure-time physical activity practice at 11 years (quartiles)	Boys^a^		Girls^a^	
	OR (95% CI)	*p*	OR (95% CI)	*p*
1st (mean 5.8 min/wk)	1.00		1.00	
2nd (mean 92.8 min/wk)	1.32 (.88, 1.98)	.18	1.02 (.76, 1.37)	.90
3rd (mean 247.5 min/wk)	1.45 (.98, 2.15)	.06	.93 (.67, 1.30)	.68
4th (mean 1,009.0 min/wk)	1.60 (1.09, 2.33)	.01	1.27 (.90, 1.80)	.17

a Adjusted for maternal and paternal schooling and family assets index.
